# Effect of Spinecor brace on pulmonary functions

**DOI:** 10.1186/1748-7161-8-S1-O46

**Published:** 2013-06-03

**Authors:** Ö Ersen, N Can, E Oguz, S Bilgic, A Sehirlioglu

**Affiliations:** 1 Gulhane Military Medical Academy, Ankara, Turkey

## Background

Brace treatment in idiopathic scoliosis is the only efficacious method of non operative treatment. The effect of dynamic SpineCor brace on pulmonary functions is not documented.

## Aim

The aim of this study is to evaluate the immediate effect of SpineCor brace on pulmonary functions.

## Methods

A total of 76 consecutive adolescent idiopathic scoliosis patients who were treated with brace included in this study. 20 of 45 patients who were treated with SpineCor brace, and 13 of 31 patients who were treated with rigid brace, were able to finish pulmonary function test (PFT). PFT is administered to 33 patients before and immediately after wearing the brace. TLC, RV, RV/TLC, FVC, FEV1, FEV1/FVC, FEF25-75 parameters of PFT are compared between SpineCor and rigid brace patients.

## Results

Average age of SpineCor group was 12.6, and average age of rigid brace group was 12.3. Cobb angle was 36.4± 7.3° in SpineCor group, and 36±6.9° in rigid. According to pulmonary function tests, there were no differences between the groups. After wearing the brace, in both groups, restrictive changes were seen, and there were no statistical difference between groups, except FEV 1 parameter (p=0,023).

## Conclusions

Although SpineCor brace is a non rigid dynamic brace, it has a similar restrictive effect on pulmonary functions like rigid braces. Further studies with larger groups are needed.
